# Metagenomic investigation of potential abortigenic pathogens in foetal tissues from Australian horses

**DOI:** 10.1186/s12864-021-08010-5

**Published:** 2021-10-02

**Authors:** Rumana Akter, Charles M. El-Hage, Fiona M. Sansom, Joan Carrick, Joanne M. Devlin, Alistair R. Legione

**Affiliations:** 1 Asia Pacific Centre for Animal Health, The Melbourne Veterinary School, The University of Melbourne, Parkville, Victoria 3010 Australia; 2 Department of Medicine (Royal Melbourne Hospital), The University of Melbourne, Parkville, Victoria 3010 Australia; 3 Equine Specialist Consulting, Scone, New South Wales 2337 Australia

**Keywords:** Abortion, Metagenomic, Zoonosis, Australia, Equine

## Abstract

**Background:**

Abortion in horses leads to economic and welfare losses to the equine industry. Most cases of equine abortions are sporadic, and the cause is often unknown. This study aimed to detect potential abortigenic pathogens in equine abortion cases in Australia using metagenomic deep sequencing methods.

**Results:**

After sequencing and analysis, a total of 68 and 86 phyla were detected in the material originating from 49 equine abortion samples and 8 samples from normal deliveries, respectively. Most phyla were present in both groups, with the exception of *Chlamydiae* that were only present in abortion samples. Around 2886 genera were present in the abortion samples and samples from normal deliveries at a cut off value of 0.001% of relative abundance. Significant differences in species diversity between aborted and normal tissues was observed. Several potential abortigenic pathogens were identified at a high level of relative abundance in a number of the abortion cases, including *Escherichia coli*, *Klebsiella pneumoniae*, *Klebsiella oxytoca, Streptococcus equi* subsp*ecies zooepidemicus, Pantoea agglomerans, Acinetobacter lwoffii*, *Acinetobacter calcoaceticus* and *Chlamydia psittaci.*

**Conclusions:**

This work revealed the presence of several potentially abortigenic pathogens in aborted specimens. No novel potential abortigenic agents were detected. The ability to screen samples for multiple pathogens that may not have been specifically targeted broadens the frontiers of diagnostic potential. The future use of metagenomic approaches for diagnostic purposes is likely to be facilitated by further improvements in deep sequencing technologies.

**Supplementary Information:**

The online version contains supplementary material available at 10.1186/s12864-021-08010-5.

## Background

Abortion in horses leads to economic and welfare losses to the equine industry, but the exact cause of an abortion event often remains unidentified [1–5]. A number of factors can result in equine abortion, which can be broadly divided into infectious and non-infectious causes. Important non-infectious causes of equine abortion include twin pregnancies, umbilical cord torsion and congenital anomalies [1, 3]. Studies investigating infectious causes of abortion have shown equid alphaherpesvirus 1 (EHV-1) to be a major viral cause, while *Streptococcus zooepidemicus* has been the most commonly identified bacterial cause [1, 5–7]. Other frequently identified bacteria include *Escherichia coli*, *Pseudomonas spp*., *Streptococcus spp*., *Enterobacter spp., Klebsiella spp., Staphylococcus spp* and *Actinobacillus spp.* [1, 5–7]. More recently the zoonotic bacterium *Chlamydia psittaci* has been identified as an important zoonotic cause of equine abortion [8–12]. Other zoonotic pathogens such as *Coxiella burnetii, Leptospira spp, Salmonella spp, Campylobacter spp* and *Toxoplasma gondii* are known to cause abortion in other species, such as cattle, sheep and goats, but are less well studied in horses [13–16].

Causes of abortion may be strongly influenced by regional differences [1, 3, 17, 18]. Knowledge of specific infectious agents present within a region can assist veterinarians to identify the cause of an abortion, but causes can change over time. This may be a reflection of improved diagnostic capabilities or the emergence or re-emergence of specific infectious agents [3, 17, 18]. Thus, monitoring the causes of equine abortion or reproductive loss is important. To date, histopathology, pathogen isolation and molecular based methods targeting known pathogens have been commonly used to detect causes of abortion in animals [1, 7, 18]. More recently metagenomic approaches have been used to successfully detect causes of abortion in cattle [19]. Metagenomics can simultaneously detect all microorganisms in clinical samples without prior knowledge of their identities. The emergence of novel pathogens, or pathogens not previously known to be present in a given region, may not be detected using targeted surveillance of known pathogens, but could be detected by metagenomic approaches [20–22]. To date metagenomic sequencing approaches have not been applied to the detection of abortigenic pathogens in equine abortion cases. The main aim of this study was to identify abortigenic pathogens in equine abortion cases in Australia using metagenomic deep sequencing methods.

## Results

### Overview of sequencing data

A total of 49 samples from equine abortion cases, 8 samples from foetal membranes from normal deliveries and 1 negative extraction control sample were analysed to investigate potential abortigenic pathogens in Australian horses. A total of 1,353,368,514 paired reads across all samples were obtained (median per sample: 20,701,371, range: 1.3 M – 55.7 M). The GC content of the reads within samples ranged from 38 to 52%. After trimming and quality filtering a total of 1,315,842,908 paired reads remained (37,625,606 removed) and were used for further analysis. After these paired reads were aligned to the horse genome to remove host associated reads a total of 32,674,936 filtered unmapped reads were used for taxonomic classification (median per sample: 324,707, range: 58,299–3.9 M). A full breakdown of reads per sample before and after filtering is available in Supplementary Table 1, found in Additional file 1.

### Pathogen communities in aborted foetal tissues and tissues from normal deliveries

A total of 68 and 86 phyla were detected at 0.001% relative abundance or higher in the material originating from the abortion cases and normal deliveries, respectively. A total of 20,156,856 reads were classified to these phyla. Approximately 2886 genera were detected in equine aborted foetal tissues and foetal membranes from normal deliveries using a cut off value of 0.001% relative abundance.

### Abundant phyla in the domain Eukaryota

The most dominant phyla in Eukaryota in both abortion cases and normal deliveries were *Ascomycota* (mean relative abundance of 26.79 and 12.75%, respectively), *Nematoda* (mean relative abundance of 1.32 and 5.45%, respectively), *Platyhelminthes* (mean relative abundance of 1.23 and 4.85%, respectively) and *Apicomplexa* (mean relative abundance of 1.07 and 4.44%, respectively).

### Abundant genera in the domain Eukaryota

In the domain Eukaryota, the most dominant genera were *Saccharomyces* (relative abundance per sample ranging from 2.08–32.82%), *Leishmania* (relative abundance per sample ranging from 2.78–23.81%) and *Plasmodium* (relative abundance per sample ranging from 3.05–6.03%). These genera were present in equine aborted foetal tissues, foetal membranes from normal deliveries and the negative extraction control sample. When re-extracted data were mapped against reference genomes to confirm the presence of these genera in the samples the results showed that mapping of *Leishmania* classified reads occurred only at the start or end point of the reference genome chromosomes (Accession numbers: NC007244-NC007287). The reads did not map to any coding regions of the genome but rather to the repeat regions at the ends of the chromosomes, suggesting they were incorrectly classified by the Centrifuge software. Similarly, no mapping to the genomes of *Saccharomyces* and *Plasmodium* was observed using re-extracted data and these were also deemed to be incorrectly classified by the Centrifuge software. Therefore, phyla/genera in the domain Eukaryota were not considered further.

### Abundant phyla in the domain Bacteria

**Fig. 1:**
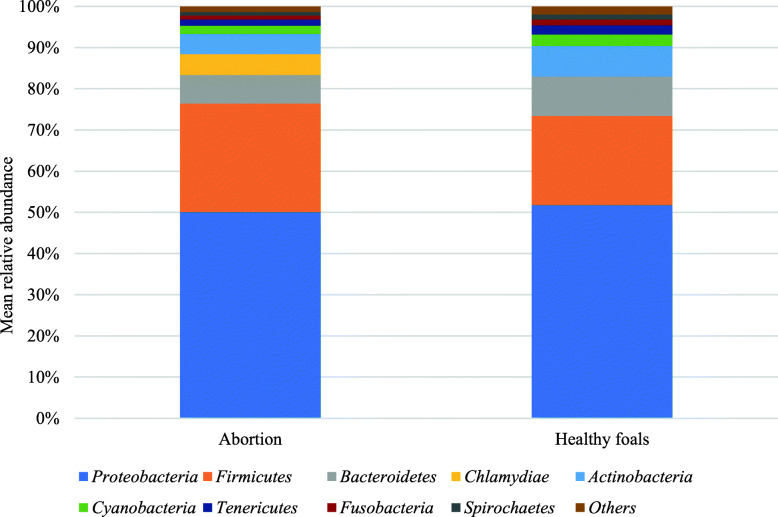
Predominant bacterial phylum present in equine aborted foetal tissues and foetal membranes from normal deliveries. Only phyla with relative mean abundance values more than 0.5% are shown. Relative mean abundance values lower than 0.5% at the phylum level were classified as Others

The average read numbers assigned to bacteria within the samples from abortion cases and normal deliveries were 46,689 and 303,810 respectively. The most dominant phyla in these groups were *Proteobacteria* (mean relative abundance of 50.06 and 51.75%, respectively) followed by *Firmicutes* (mean relative abundance of 26.37 and 21.62%, respectively), *Bacteroidetes* (mean relative abundance of 6.95 and 9.62%, respectively), *Chlamydiae* (mean of 4.99% in aborted foetal tissues) and *Actinobacteria* (mean relative abundance of 4.93 and 7.39%, respectively) (Fig. 1).

### Abundant genera in the domain Bacteria

**Fig. 2:**
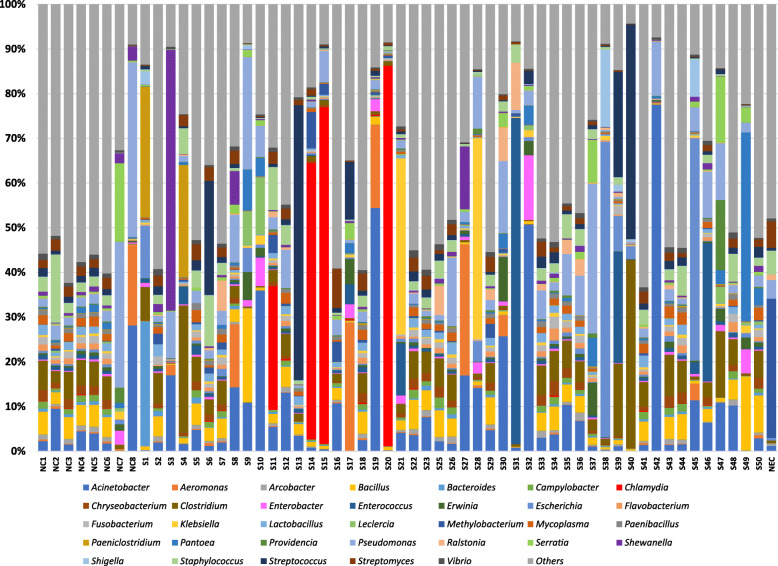
Predominant genera of bacteria present in equine aborted foetal tissues and foetal membranes from normal deliveries. Genera with relative abundances of > 0.5% are shown. Genera with relative abundances values less than 0.5% were classified as Others. NC1 to NC8 = samples from normal deliveries. S1 to S50 = samples from abortion cases. NEC = extraction control

**Fig. 3:**
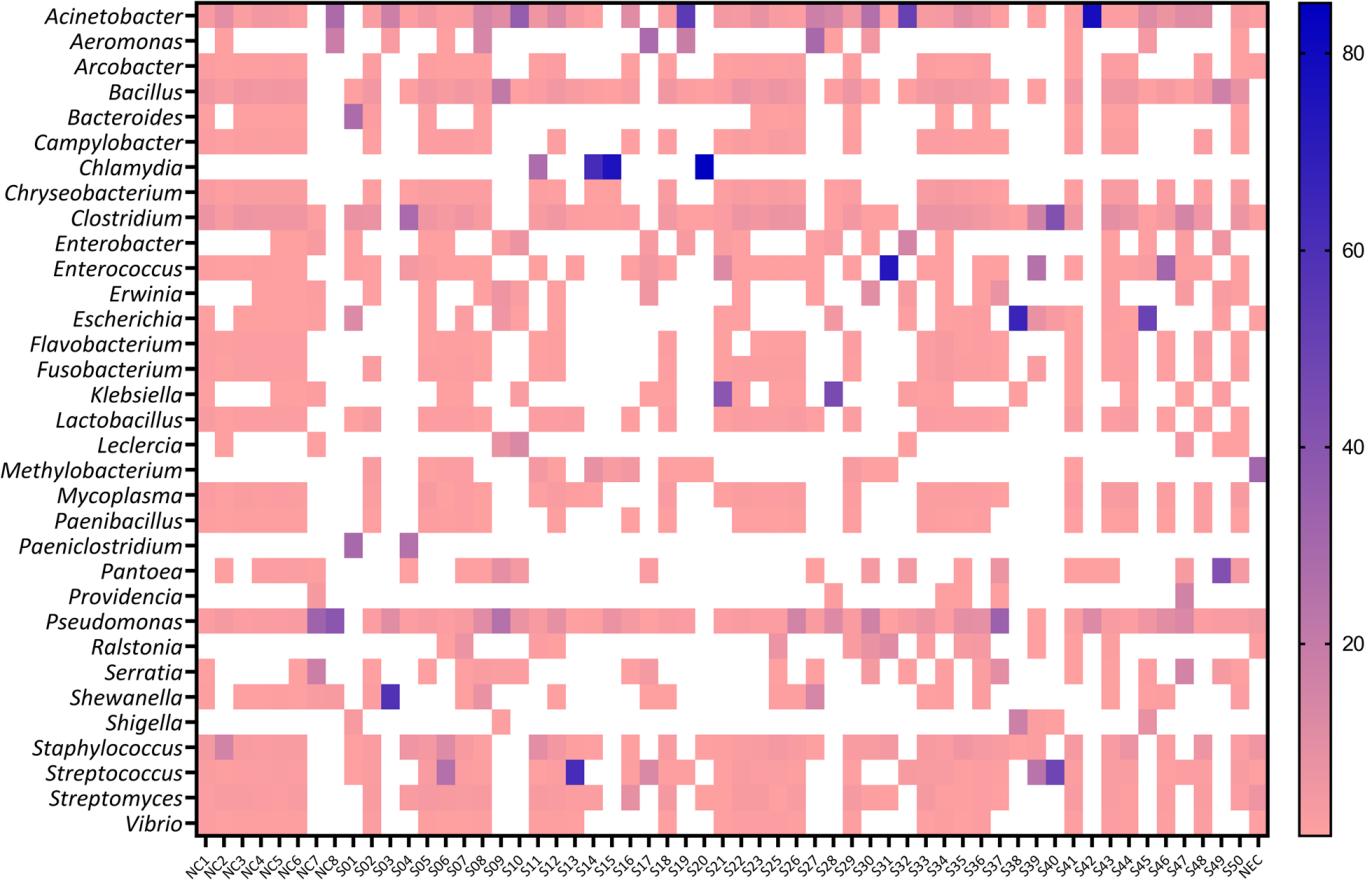
Heat map showing the relative abundance levels of the most abundant bacterial genera in aborted foetal tissues and foetal membranes from normal deliveries. Relative abundances results > 0.5% are shown. NC1 to NC8 = samples from normal deliveries. S1 to S50 = samples from abortion cases. NEC = extraction control

Abundant genera are shown in Figs. 2 and 3. The genus *Chlamydia* was dominant in four abortion cases (relative abundance per sample ranging from 27.67–85.10%). *Acinetobacter* was the most abundant genera in at least seven aborted foetal tissues (relative abundance per sample ranging from 10.73–77.56% per sample). Others dominant genera present in abortion cases were *Paeniclostridium, Bacteroides*, *Shewanella*, *Clostridium*, *Pseudomonas*, *Streptococcus*, *Bacillus*, *Aeromonas*, *Klebsiella*, *Enterococcus*, *Escherichia* and *Pantoea*. In the foetal membranes from normal deliveries the most abundant genera included *Pseudomonas*, *Serratia, Acinetobacter* and *Aeromonas* (Figs. 2 and 3). A breakdown of all genera that were present at greater than 0.001% relative abundance can be found in Additional file 2.

### Abundant genera in the domain viruses

The most abundant genus of virus was *Agricanvirus* (relative abundance of 0.6–8.11% per sample).

### Abortigenic species present in samples

Several potentially abortigenic bacteria were detected at high relative abundance levels (> 5%) in the abortion cases including *C. psittaci, E. coli, Klebsiella pneumoniae, Klebsiella oxytoca*, *Streptococcus equi* subsp. *Zooepidemicus, Acinetobacter lwoffii* and *Acinetobacter calcoaceticus* (Table 1). None of these bacteria were detected at relative abundance levels > 5% in non-abortion cases. These reads classified to these species were mapped to an appropriate reference genome to confirm that the reads were correctly classified.

**Table 1:** Potentially abortigenic bacteria species detected at a relative abundance greater than 5%

Species	Relative Abundance values per sample (%)															
	S1	S9	S11	S12	S14	S15	S20	S21	S28	S30	S38	S39	S40	S42	S45	S49
***Escherichia coli***	11.02	–	–	–	–	–	–	–	–	–	63.00	–	–	–	46.25	–
***Chlamydia psittaci***	–	–	26.17	–	59.55	64.40	84.10	–	–	–	–	–	–	–	–	–
***Klebsiella pneumoniae***	–	–	–	–	–	–	–	–	29.92	–	–	–	–	–	–	–
***Klebsiella oxytoca***	–	–	–	–	–	–	–	13.89	–	–	–	–	–	–	–	–
***Streptococcus equi subsp. zooepidemicus***	–	–	–	–	–	–	–	–	–	–	–	16.48	31.20	–	–	–
***Pantoea agglomerans***	–	5.40	–	–	–	–	–	–	–	–	–	–	–	–	–	18.08
***Acinetobacter lwoffii***	–	–	–	5.84	–	–	–	–	–	5.14	–	–	–	13.43	–	–
***Acinetobacter calcoaceticus***	–	–	–	–	–	–	–	–	–	–	–	–	–	5.69	–	–

### Analysis of microbial profiles

**Fig. 4:**
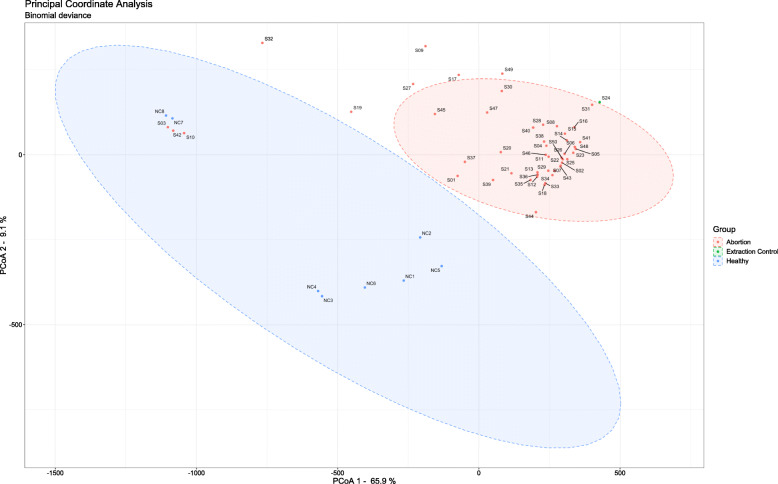
Orientation of the microbial profiles of bacterial species from abortion cases and normal (healthy) deliveries by principal coordinates analysis (PCoA)

Visualising our principal coordinates analysis (PCoA) (Fig. 4) identified strong clustering of most abortion samples, with a small cluster of three abortion samples with two healthy foal samples (NC7 and NC8). A Permutational ANOVA of the species dissimilarity matrix used to generate this plot highlighted significant separation of the microbial profiles of abortion and non-abortion groups (Permutations = 999, F = 7.4106, *P* < 0.001). Whilst pairwise PERMANOVAs corrected for multiple comparisons identified significant separation of the centroids between the Chlamydia and Healthy groups (Permutations = 999, F = 12.271, Adjusted *P* = 0.011) and Unknown and Healthy groups (Permutations = 999, F = 15.346, Adjusted *P* = 0.011).

**Fig. 5:**
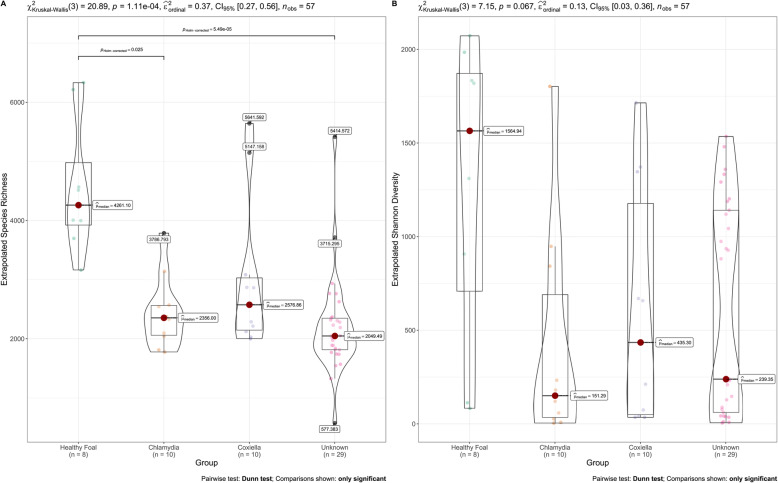
Diversity analysis: Extrapolated Hill numbers representing species richness (**A**) and diversity (**B**) of abortion and non-abortion groups, with the former further divided into samples known to be PCR positive for *C. psittaci* (*Chlamydia*), samples known to be PCR positive for *C. burnetii* (*Coxiella*) and samples known to be PCR negative for selected abortigenic pathogens (Unknown)

Marked variation between the number of reads classified as bacteria between samples (ranging from 492 to 1,490,220) was observed (Additional file 1). To compare diversity, extrapolated Hill numbers were assessed to account for between sample read number variation [23]. Significant differences were identified when comparing Hill numbers equivalent to extrapolated species richness (χ^2^ = 20.89, *P* = < 0.001) but not extrapolated Shannon diversity (χ^2^ = 7.15, *P* = 0.067) (Fig. 5). Within the extrapolated species richness values there was a significant difference between healthy foals and the *Chlamydia* positive group (adjusted *P* = 0.025), and between healthy foals and foals with unknown causes of abortion (adjusted *P* < 0.001), with healthy foals having a higher richness in both cases.

### Virulence factors and antibiotic resistance genes in samples

The presence of virulence genes from bacterial samples was assessed using both short read mapping and contig screening of assembled sequences. The mapping method utilised both merged reads from all abortion samples and individual read datasets. In the case of merged reads, virulence factors were found mostly for *A. baumannii* (OmpA)*, E. coli* (fimB, eprI, cdiB and nipI) and *Pseudomonas fluorescens* (algU, flgG, fliN, pilJ, pilG, fleQ, flhA, fleN and algD). These are shown in more detail in Table 2. Individual mapping revealed two abortion foals (S01 and S03) with sufficient read depth to identify virulence factors, with virulence factors from *E. coli* (*eprI*) and *A. baumannii* (IS4 family transposase ORF 1) being identified in these samples respectively. Interestingly, NC7 and NC8 both had substantially more virulence factors identified, totalling 131 and 84, respectively. This was reflected in classification of metagenomic assembled contigs using abricate [24], which identified no virulence factors in abortion samples, but 19 and 3 virulence factors in NC7 and NC8, respectively. Inspection of the intermediate mapping files identified virulence genes with at least 50% gene coverage in 11 abortion samples (Additional file 3).

**Table 2:** Virulence factors presence in samples determined using SRST2 mapping against the Virulence Factor Database

Reference	Gene	Coverage	Depth	Divergence	Length	Organism	Description
VFG038174	ompA	92.0	17.2	8.6	1062	*Acinetobacter baumannii* AB0057	outer membrane protein A [Outer membrane protein (CVF776)]
VFG012267	fimB	90.7	2.8	2.0	603	*Escherichia coli* 536	type 1 fimbriae regulatory protein FimB [Type I fimbriae (CVF426)]
VFG019910	algU	92.6	3.0	7.1	582	*Pseudomonas fluorescens* SBW25	alternative ECF subfamily sigma factor [Alginate regulation (CVF523)]
VFG033163	fimB	90.7	2.8	0.7	603	*Escherichia coli* O55:H7 str. CB9615	type 1 fimbriae regulatory protein fimB [Type I fimbriae (CVF426)]
VFG033162	fimB	90.7	2.8	2.0	603	*Escherichia coli* O127:H6 str. E2348/69	tyrosine recombinase [Type I fimbriae (CVF426)]
VFG038010	ABZJ_00085	92.5	3.5	1.7	570	*Acinetobacter baumannii* MDR-ZJ06	IS4 family transposase ORF 1 [Capsule (CVF775)]
VFG033166	fimB	90.7	2.8	1.1	603	*Escherichia coli* O157:H7 str. TW14359	tyrosine recombinase [Type I fimbriae (CVF426)]
VFG033169	fimB	90.7	2.8	0.5	603	*Escherichia coli* O111:H- str. 11,128	tyrosine recombinase/inversion of on/off regulator of fimA [Type I fimbriae (CVF426)]
VFG042090	eprI	97.5	2.1	0.0	240	*Escherichia coli* O157:H7 str. Sakai	EprI [ETT2 (SS017)]
VFG014223	flgG	90.5	2.1	5.3	786	*Pseudomonas fluorescens* Pf0–1	hypothetical protein [Flagella (CVF521)]
VFG033168	fimB	90.7	2.8	0.2	603	*Escherichia coli* O103:H2 str. 12,009	tyrosine recombinase/inversion of on/off regulator of fimA [Type I fimbriae (CVF426)]
VFG033178	fimB	90.7	2.8	0.2	603	*Escherichia coli* O7:K1 str. CE10	tyrosine recombinase/inversion of on/off regulator of fimA [Type I fimbriae (CVF426)]
VFG014522	fliN	90.4	2.9	3.6	459	*Pseudomonas fluorescens* Pf0–1	flagellar motor switch protein [Flagella (CVF521)]
VFG014052	pilJ	95.0	3.3	7.5	2061	*Pseudomonas fluorescens* Pf0–1	chemotaxis sensory transducer [Type IV pili twitching motility related proteins (CVF519)]
VFG014013	pilG	91.6	2.5	9.4	405	*Pseudomonas fluorescens* Pf0–1	Response regulator receiver domain protein (CheY) [Type IV pili twitching motility related proteins (CVF519)]
VFG014366	fleQ	92.3	2.7	5.9	1476	*Pseudomonas fluorescens* Pf0–1	Sigma-54 Specific Transcriptional Regulator, Fis family [Flagella (CVF521)]
VFG012264	fimB	90.7	2.8	0.9	603	*Escherichia coli* O157:H7 str. EDL933	recombinase involved in phase variation; regulator for fimA [Type I fimbriae (CVF426)]
VFG033181	fimB	90.7	2.8	1.8	603	*Escherichia coli* O83:H1 str. LF82	Type 1 fimbriae regulatory protein fimB [Type I fimbriae (CVF426)]
VFG036040	cdiB	96.5	1.8	0.3	312	*Escherichia coli* str. Clone D i2	hypothetical protein [Contact-dependent inhibition CDI system (CVF747)]
VFG000477	rpoS	98.6	3.3	5.0	993	*Salmonella enterica* subsp. enterica serovar Typhimurium str. LT2	sigma S (sigma 38) factor of RNA polymerase, major sigmafactor during stationary phase [RpoS (VF0112)]
VFG038379	ASA_2470	94.4	1.7	5.3	504	*Aeromonas salmonicida* subsp. salmonicida A449	hypothetical protein [T6SS (CVF782)]
VFG014915	algU	93.0	3.0	7.2	582	*Pseudomonas fluorescens* Pf-5	RNA polymerase sigma-70 factor [Alginate regulation (CVF523)]
VFG014600	flhA	90.4	3.1	6.5	2130	*Pseudomonas fluorescens* Pf0–1	flagellar biosynthesis protein [Flagella (CVF521)]
VFG014625	fleN	90.1	2.3	8.2	822	*Pseudomonas fluorescens* Pf-5	flagellar synthesis regulator FleN [Flagella (CVF521)]
VFG014916	algU	92.1	3.1	7.3	582	*Pseudomonas fluorescens* Pf0–1	RNA polymerase sigma-70 factor [Alginate regulation (CVF523)]
VFG033175	fimB	90.7	2.8	0.2	603	*Escherichia coli* O78:H11:K80 str. H10407	tyrosine recombinase/inversion of on/off regulator of fimA [Type I fimbriae (CVF426)]
VFG038025	ABZJ_00086	93.3	3.1	1.0	447	*Acinetobacter baumannii* MDR-ZJ06	IS4 family transposase ORF 2 [Capsule (CVF775)]
VFG033170	fimB	90.7	2.8	0.4	603	*Escherichia coli* O26:H11 str. 11,368	tyrosine recombinase [Type I fimbriae (CVF426)]
VFG033171	fimB	90.7	2.8	0.4	603	*Escherichia coli* O7:K1 str. IAI39	tyrosine recombinase [Type I fimbriae (CVF426)]
VFG043469	SSU98_1513	95.5	1.9	6.9	1308	*Streptococcus suis* 98HAH33	phosphopyruvate hydratase [Fibronectin-binding protein (AI215)]
VFG043545	ECS88_3547	91.5	4.3	4.7	885	*Escherichia coli* O45:K1:H7 str. S88	lipoprotein NlpI [NlpI (AI331)]
VFG000871	fimB	90.7	2.8	2.0	603	*Escherichia coli* CFT073	Type 1 fimbriae Regulatory protein fimB [Type 1 fimbriae (VF0221)]
VFG012263	fimB	90.7	2.8	0.5	603	*Escherichia coli* str. K^−12^ substr. MG1655	tyrosine recombinase/inversion of on/off regulator of fimA [Type I fimbriae (CVF426)]
VFG014756	algD	93.5	3.0	6.9	1317	*Pseudomonas fluorescens* Pf0–1	UDP-glucose/GDP-mannose dehydrogenase [Alginate biosynthesis (CVF522)]

Merged mapping analysis identified a single antibiotic resistance gene, Streptomyces B (strB) from the abortion cases. Individual mapping failed to confidently identify any antibiotic resistance genes (gene coverage > 90%) in aborted foals, but did identify sulI, aadA, and DfrA5 in NC7 (conferring resistance to sulphonamides, aminoglycosides, and trimethoprim), and *sulII* in NC8 (conferring sulphonamide resistance). Inspection of the intermediate mapping files identified additional resistance genes with at least 50% gene coverage in samples S42 (including the strB allele), NC7, and NC8 (Additional file 3). Metagenomic assembly and matching using the tool abricate identified blaOXA-278 in S42, blaOXA-549 and catB11 (Chloramphenicol resistance) in S03, mcr9.1 in NC7 and blaOXA-12 in NC8.

Table 2: Virulence factors presence in samples determined using SRST2 mapping against the Virulence Factor Database.

## Discussion

Several studies have investigated equine microbiota of the intestine [25], hindgut [26–29], uterus [30, 31], placenta [29] and respiratory tract [32] but the microbiota of equine aborted foetal tissues has not previously been investigated.

Although the uterus is not sterile [33] there is some debate over the sterility of the foetus and placenta [34–37]. Thus, ascribing associations between the detection of bacterial DNA and abortion is not straightforward. This study used samples from aborted equine foetuses, as well as foetal membranes from healthy deliveries, to compare the microbiota present in abortion and non-abortion cases. The material from healthy deliveries necessarily excluded foetal tissues (lung, liver, spleen) that were included in the aborted samples. This should be considered during the interpretation of the results. A high level of abundance at a genus or species level potentially indicates a high bacterial load and may suggest that the infection is of clinical significance and is potentially linked to the abortion [19]. All high abundance eukaryotic classifications, when investigated at a read level, were found to be erroneous classifications based on matching of reads to repeat regions in eukaryotic reference genomes. Similarly there was limited detection of viruses of interest, with the majority of virus hits matching to bacteriophage. This is not unexpected due to the long-term storage of samples at − 80 °C without preservation solutions, which may not have been conducive to preservation of viral DNA, and the comparably small genomes of viruses leading reduced detection of any DNA that is present compared to host or bacteria. For these reasons, our study examined the relative abundance of different taxonomic groups of bacteria, focussing on those that had a high relative abundance. If next generation sequencing is used in future for investigating equine abortions, using suitable nucleic acid storage reagents will be critical for obtaining high quality output, in addition to utilising RNA sequencing methodologies to gain information on the presence of abortogenic RNA viruses.

The most abundant taxonomic groups of bacteria in the samples were *Proteobacteria, Firmicutes*, *Bacteroidetes*, *Chlamydiae* and *Actinobacteria*. Besides the detection of *Chlamydiae* (detected in abortion samples only) these findings are similar to a previous report investigating abortion samples and healthy placentas from cattle using 16S amplicon sequencing, where the most abundant phyla of bacteria were *Proteobacteria*, *Firmicutes* and *Bacteroidetes* [19]. The results of our primary output of SRST2 and abricate were reflective of the relative number of reads classified to each bacterial species. As both methods require high read numbers, either to obtain > 90% gene coverage across a bacterial genome (and by extension, each gene therein) for SRST2 or for metagenomic assembly of contigs for abricate, we obtained limited hits when investigating individual samples. Examination of intermediate files of SRST2 allowed us to investigate virulence and antibiotic resistance gene detection per sample, whilst utilising the merged dataset gave a greater level of confidence that these genes occurred within our samples.

Some genera of bacteria were present only in the abortion samples or were present at a higher level of relative abundance in the abortion samples compared to non-abortion samples. The genus *Chlamydia* was the dominant bacterial genera in at least four abortion samples and was not found in non-abortion samples. The only abundant species under this genus was *C. psittaci.* Several studies have reported *C. psittaci* as the cause of equine abortion globally [8, 10–12]. The detection of *C. psittaci* in abortion samples only and at a high relative abundance (26.17–84.10%) is consistent with *C. psittaci* being a cause of equine abortion.

Besides *Chlamydia,* other dominant genera present in abortion cases were *Streptococcus*, *Klebsiella*, *Escherichia* and *Pantoea.* These genera of bacteria can be sporadic causes of equine abortion and are often associated with ascending infections that infect the placenta and foetus via the transcervical route. *Streptococcus* was the most dominant genus in three abortion cases. *S. equi subsp. zooepidemicus* was detected in two of these cases (relative abundance levels of 16.48 and 31.20%) and *S. parauberis* was dominant in one case (relative abundance of 11.88%). *S. equi subsp. zooepidemicus* is a common bacteria detected in equine abortion cases [1, 5, 6]. This bacteria usually inhabits in the lower genital tract of mares and can enter into the placenta and foetus resulting in placentitis and abortion [6]. The abortogenic potential of *S. parauberis* is undescribed.

The genus *Klebsiella* was abundant in two equine abortion cases where *K. pneumoniae* (*relative abundance of* 29.92%) and *K. oxytoca* (*relative abundance of* 13.89%) were the most dominant species. *Klebsiella sp* are abundant in the environment and are a component of the normal equine urogenital and intestinal microflora [38, 39]. Both *K. pneumoniae* and *K. oxytoca* are known causes of abortion in mares [5, 40, 41].

*E. coli* was predominant in three abortion cases (*relative abundance levels of* 11.02, 46.25 and 63.00%) while *P. agglomerans* was most abundant in two abortion cases (*relative abundance levels of* 5.40 and 18.08%). *E. coli* and *P. agglomerans both belong to the Enterobacteriaceae* family and have previously been isolated from equine abortion materials [5, 8, 42]. The *Escherichia* spp. fimB virulence gene, a type 1 fimbriae regulatory protein, was identified in our virulence factor analysis. Type 1 fimbriae are the most common and well categorized of the enterobacterial adhesive surface organelles [43]. Most *E. coli* strains as well as other members of the *Enterobacteriaceae family* have type 1 fimbriae which play a significant role in colonizing host tissues [43–45]. A link between urinary tract pathogenesis and the adhesion conferred by type 1 fimbriae in an *E. coli* strain has been demonstrated [46, 47]. The presence of this *Escherichia spp* virulence gene could suggest that the *E. coli* detected in this study were capable of causing disease.

*The genus Acinetobacter was detected in abortion and non-abortion cases, but the higher relative abundance levels in abortion cases suggests Acinetobacter could be a potential cause of abortion. Acinetobacter lwoffii* was the most abundant bacteria in three abortion cases *(relative abundance values of 5.14, 5.84 and 13.32%) and A. calcoaceticus was the most abundant bacteria in another abortion case (relative abundance of 5.69%). In humans Acinetobacter* can cause nosocomial opportunistic infections [48, 49], can often be detected in intra-amniotic infection cases and can potentially inhabit the placenta [34]. *Adverse pregnancy outcomes, including perinatal death*, have been reported [50–53]. In horses, *Acinetobacter* can cause wound infections, septicaemia, bronchopneumonia, neonatal encephalopathy and eye infections [54]. *Acinetobacter* has also been isolated from clustered cases of equine abortion and equine amnionitis and foetal loss in NSW, Australia [55]. *A. calcoaceticus,* and *A. lwoffii have been detected in aborted materials from horses* [52], *buffalo* [53] *or cattle* [50]. The presence of the *Acinetobacter* spp. *ompA* gene, a virulence factor, suggests that the *Acinetobacter* detected in this study may have been capable of causing disease. This virulence factor is associated with regulation of the adhesion, aggressiveness, and biofilm formation of *A. baumannii*. The mortality rate of nosocomial pneumonia and bacteraemia caused by *Acinetobacter* spp. can be influenced by the excessive production of *OmpA* [56].

The contamination of samples from the environment after abortion or parturition, as well as contamination during processing, should be considered when interpreting the results from this study. Environmental or commensal bacteria such as *Pseudomonas, Methyl bacterium* and *Clostridium,* which can be associated with contamination of samples from extraction kits and the environment [57],were found in abortion and non-abortion samples, as well as the negative extraction control sample. Placental tissues are susceptible to the contamination of bacterial DNA after abortion or parturition, thus the presence of contaminating bacteria in the placental sample is not surprising [35]. The presence of such environmental bacteria in the samples can impede the ability to identify other pathogens [58].

The PCoA showed diverse taxonomic profiles between the abortion and non-abortion groups, with the strong clustering shown in the two dimensions accounting for more than 70% of the total data variability. Extrapolated diversity analyses highlighted that the species richness in abortion cases was lower than in non-abortion cases. Furthermore, significant differences in species richness were detected between non-abortion cases and abortion cases known to be PCR positive for *C. psittaci*. Whilst extrapolated diversity metrics were not significantly different, there still appeared to be a biological difference that may bear out with increased sample sizes. It is possible that the overgrowth of pathogenic bacteria leads to a reduced diversity [59], which may explain the reduced richness of species in abortion samples. Antibiotic treatment may also reduce bacterial richness, however the details of any antibiotic treatments used in the mares in this study are unknown. Interestingly two samples in the ‘healthy’ group, NC7 and NC8, had high read numbers and both virulence and antibiotic-resistance genes were identified in these samples. Additionally, via our diversity analysis it was identified that they clustered more closely with S03, S42, and S10 than they did the rest of the ‘healthy’ foal group samples, suggesting they share a similar array of bacterial species. The exact reason for this clustering, and the abundance of bacterial taxa in these healthy born placental samples is unclear. It may, as outlined above, be reflective of placental contamination after parturition.

Deep sequencing technologies are rapidly advancing and are likely to improve further [60] but limitations still exist. In this study metagenomics did not detect *C. burnetii* in samples known to be PCR-positive for this pathogen, albeit at relatively low levels [16], indicating the sensitivity of the metagenomic approach is not as good as targeted qPCR for this bacterium. Similar limitations may be present for the sensitive detection of other pathogens. These issues may be mitigated in future by utilising appropriate storage reagents designed for preserving samples for metagenomic sequencing. Likewise the use of chemicals to degrade host DNA and thus increase the proportion of DNA matching to non-eukaryotic species would be ideal [61]. Marrying traditional culture-based methods to complement metagenomic detection may be appropriate in the short term, however continued improvements in sequencing technologies the future use of deep sequencing approaches is likely to assist with identifying new causes of equine abortion and could be used as a diagnostic tool that would avoid the need to test for multiple pathogens using targeted approaches.

## Conclusions

Although metagenomic approaches have previously been used as a molecular diagnostic tool to detect causes of infectious diseases, such approaches have not previously been applied to the detection of causes of equine abortion. In this study several potential equine abortigenic pathogens were detected using metagenomics, showcasing the ability of metagenomic approach to detect multiple agents in equine abortion samples. It is possible that metagenomics may have diagnostic applications for equine abortion cases in the future.

## Methods

### Samples collection and initial screening

Samples from equine abortion cases were submitted to our diagnostic laboratories at the Melbourne Veterinary School between 1994 to 2019. The samples originated from New South Wales and Victoria (VIC), Australia. Metadata for each sample is available in Supplementary Table 2 in Additional file 1. Foetal tissues including lung, liver, spleen, and thymus, as well as foetal membranes were submitted for each case. The tissues were stored at − 80 °C in 1.5 mL tubes after submission. Selected samples were thawed, and a plastic-shafted rayon tipped swab (Copan Italia) was used to sample each tissue. Swabs from tissue originating from the same foetus were combined in 500 μL of PBS (pH 7.4) and the pooled swabs were stored at − 80 °C until DNA extraction. Each tube of swab/PBS solution was vortexed for approximately 5 s before a 200 μL aliquot was removed for extraction. DNA was extracted from the PBS solution by a Kingfisher robot with a MagMAX™ Core Nucleic Acid Purification Kit (Thermo Fisher Scientific) according to the manufacturer instructions. Extracted DNA was eluted in 90 μL of elution buffer and stored at − 80 °C for further use.

DNA extracted from each sample was to screen the samples for equine herpesviruses [62], *Chlamydiaceae* [63], *C. burnetii* [64], *Leptospira* spp. [65] and *Toxoplasma* spp. [66] as previously described [16]. A total of 49 abortion cases (pooled lung, spleen, thymus, and foetal membranes for each foetus) were then selected for this study. Of these, 10 samples were positive for *C. burnetii* DNA by qPCR (samples S1 – S10 in this study), 10 samples were positive for *C. psittaci* (samples S11 – S20 in this study) and 29 samples were neither positive for *C. burnetii*, *C. psittaci* or herpesviruses (samples S21, S22, S23 and S25- S50 in this study). A negative extraction control (S24) was added with the sample. DNA extracted from equine foetal membranes originating from 8 normal delivery foals were also included in this study (samples NC1 – NC8) and were processed as described above. These samples were from full term deliveries that required no intervention. The foals from these deliveries all stood within an hour and sucked within 2 hours. All had serum IgG levels > 800 mg/dL at 24 h and the placenta has no gross pathology. One negative extraction control (PBS) was also included in the study.

### DNA library preparation and sequencing

Following extraction, the DNA concentration of the samples was measured using a 4200 TapeStation system (Agilent Technologies). DNA libraries were prepared using Illumina Truseq DNA library preparation kit according to the manufacturer’s instructions at Walter and Eliza Hall Institute (WEHI, Australia). Sequencing was performed using 150 cycle mid kit on Illumina NextSeq 500 platform to produce paired end reads of 150 bp (2 × 75 bp) at WEHI, Australia.

### Data analysis

FastQC Version 0.11.8 [67] was used to check the quality of the Illumina reads. Reads were then trimmed to remove low-quality ends and to remove Truseq adapters using TrimGalore Version 0.6.4 [68] using the following criteria: low-quality ends (quality score below 25) were trimmed from reads, along with adapter sequences. Reads with an error rate greater than 0.1 were discarded. Reads that became shorter than 20 bp were discarded and unpaired single-end reads less than 35 bp were discarded. FastQC was used to confirm the removal of adapters and to assess the post-trimming quality of the reads. After trimming, paired reads were mapped to the horse genome reference assembly EquCab3.0 (Genbank Accession Number: GCF_002863925.1) [69] using minimap2 version 2.15 programme [70]. Then paired reads were filtered using Samtools 1.9 version [71] and unmapped reads were used for taxonomic classification using NCBI nt database [72] by Centrifuge version 1.0.4 [73]. Centrifuge analyses were performed on the University of Melbourne High Performance Computer Cluster ‘Spartan’ [74].

Centrifuge outputs were visualised and analysed using pavian tools [75] in Rstudio Software. Species with a relative abundance of less than 0.001% across all samples were removed from the dataset. The richness and diversity of species in samples were analysed using extrapolated Hill numbers [23], which were generated using the R package iNext [76]. The differences in species richness and diversity between abortion samples and normal delivery samples was assessed using the Kruskal-Wallis test with the Dunn test for multiple comparisons within the package ggstatsplot [77]. Dunn test is a variant of the Tukey test utilised when only a small number of pairwise comparisons are utilised. *P*-values were adjusted for multiple comparisons using Holm method [78]. Differences were considered statistically significant if the adjusted *P* value was < 0.05. A heat map of relative abundance data was generated using GraphPad Prism8 Software. A diversity matrix and principal coordinates analysis was undertaken using the R package vegan [79] to compare the diversity of the microbial communities between samples and identify any clustering. Due to the uneven nature of metagenomic sequencing we utilised the binomial deviance method of assessing community distances as it is more suitable when working with uneven sample sizes [80]. Statistical assessment of dissimilarity between abortion and healthy samples was conducted using the Permutational Multivariate Analysis of Variance (PERMANOVA) function ‘adonis2’ in vegan, using 999 permutations [81], whilst pairwise PERMANOVAs were conducted between the subgroups of Chlamydia positive, Coxiella positive, healthy births, and abortions of unknown cause. Correction for multiple comparisons was undertaken using the Holm method as described above.

### Taxonomic classification validation

Validation of the taxonomic classification analyses was undertaken for all species with a high relative abundance (> 5%) as determined using Centrifuge classification. To achieve this, all reads associated with the genus of the identified species were extracted from the dataset using the ‘re-extract’ function of Recentrifuge [82]. These reads were then mapped to an appropriate reference genome of that species using bowtie 2 [83] to confirm that the classified reads mapped to the genome as expected.

### Antibiotic resistance and virulence gene identification

The read mapping tool SRST2 [84] and the contig screening tool abricate [24] were used to identify the presence of antibiotic resistance genes and virulence genes in the dataset. Read mapping was undertaken using individual sample reads, and merged reads from all abortion samples, mapped against the Virulence Factor Database [85] and the ARG-ANNOT database [86]. Merged reads were utilised to improve the coverage and depth of hits against database genes due to the likely low coverage in the metagenomic sample pool. In addition to genes reported in SRST2 output, which require > 90% coverage and > 1 depth, genes with > 50% coverage were extracted from SRST2 intermediate files. For contig screening, unfiltered trimmed reads from each sample were assembled using megahit [87] and screened against both the ARG-ANNOT database and the Virulence Factor Database.

## Supplementary Information

**Additional file 1: Supplementary tables.** Two supplementary tables including additional data for the manuscript, including 1) Total reads for each sample, before and after filtering; 2) Sample metadata.

**Additional file 2.** Bacterial genera. Breakdown of the relative abundance of all bacterial genera identified in samples through Centrifuge taxonomic classification used to generate Fig. 2

**Additional file 3.** Virulence and antimicrobial resistance genes. Tables of results from SRST2 and abricate analysis of metagenomic data against the ARGANNOT and VFDB databases

## Data Availability

Raw data that support the findings of this study have been deposited in Genbank sequence read archive under the accession number PRJNA664255 (https://www.ncbi.nlm.nih.gov/sra/PRJNA664255).

## Abbreviations

ANOVA: Analysis of Variance
DNA: Deoxyribonucleic acid
EHV-1: Equid alphaherpesvirus 1
IgG: Immunoglobulin G
NSW: New South Wales
PBS: Phosphate buffered saline
PCoA: Principal coordinates analysis
PCR: Polymerase chain reaction
PERMANOVA: Permutational Analysis of Variance
qPCR: quantitative polymerase chain reaction
RNA: Ribonucleic acid
VIC: Victoria
WEHI: Walter and Eliza Hall Institute
