# Supplementary material for: Metagenomic investigation of potential abortigenic pathogens in foetal tissues from Australian horses
Source: BMC Genomics. 2021 Oct 2;22:713. doi: 10.1186/s12864-021-08010-5 (PMC8487468; doi:10.1186/s12864-021-08010-5)
Supplement: Supplementary file 1 — Additional file 1: Supplementary tables. Two supplementary tables including additional data for the manuscript, including 1) Total reads for each sample, before and after filtering; 2) Sample metadata. [file 12864_2021_8010_MOESM1_ESM.docx]

**Supplementary Table 1.** An overview of the sequencing metrics of each of the samples included in the study.

| **Sample id** | **Total paired reads before quality control** | **GC%** | **Total paired reads after quality control** | **GC%** | **Total paired reads removed by quality control** | **Reads after filter against Equine genome** | **Total classified reads** | **Total Reads Classified as bacteria** |
| --- | --- | --- | --- | --- | --- | --- | --- | --- |
| S1 | 29805194 | 42 | 29563060 | 42 | 242134 | 386568 | 294117 | 17293 |
| S2 | 24066348 | 42 | 23735424 | 42 | 330924 | 240659 | 189065 | 756 |
| S3 | 28588292 | 43 | 27592380 | 43 | 995912 | 3947451 | 2258473 | 1131948 |
| S4 | 20449426 | 48 | 19735534 | 48 | 713892 | 402706 | 351040 | 2865 |
| S5 | 14947794 | 43 | 14055290 | 43 | 892504 | 167564 | 132401 | 523 |
| S6 | 16315220 | 47 | 15913338 | 47 | 401882 | 280405 | 240847 | 894 |
| S7 | 24927272 | 46 | 24015840 | 46 | 911432 | 491119 | 427555 | 961 |
| S8 | 14001030 | 46 | 13552188 | 46 | 448842 | 177168 | 140658 | 912 |
| S9 | 23390740 | 44 | 22239190 | 44 | 1151550 | 357075 | 281691 | 26724 |
| S10 | 19332800 | 46 | 18853064 | 46 | 479736 | 862115 | 608482 | 183875 |
| S11 | 29199424 | 48 | 27901024 | 48 | 1298400 | 625482 | 558835 | 1499 |
| S12 | 21777830 | 41 | 20617198 | 41 | 1160632 | 274601 | 211764 | 1291 |
| S13 | 26167330 | 44 | 25708912 | 44 | 458418 | 321455 | 257978 | 2742 |
| S14 | 12742680 | 45 | 12538852 | 45 | 203828 | 209607 | 174611 | 1604 |
| S15 | 12047744 | 41 | 11822550 | 41 | 225194 | 163223 | 128642 | 2811 |
| S16 | 13281768 | 49 | 12858270 | 49 | 423498 | 197851 | 164227 | 829 |
| S17 | 23797552 | 46 | 22969130 | 46 | 828422 | 396234 | 330815 | 17841 |
| S18 | 28912784 | 44 | 27873864 | 44 | 1038920 | 414568 | 331424 | 1091 |
| S19 | 25287122 | 44 | 24956972 | 44 | 330150 | 448424 | 348121 | 26920 |
| S20 | 27186144 | 42 | 26597826 | 42 | 588318 | 377826 | 305912 | 10844 |
| S21 | 29014090 | 39 | 27635022 | 39 | 1379068 | 325635 | 251433 | 3496 |
| S22 | 20777352 | 40 | 20073354 | 40 | 703998 | 214038 | 161973 | 820 |
| S23 | 12344244 | 41 | 12128988 | 41 | 315256 | 158167 | 122141 | 571 |
| S24 | 1298862 | 94 | 524564 | 94 | 774298 | 204800 | 203394 | 191 |
| S25 | 16501152 | 39 | 15916902 | 39 | 584250 | 147177 | 109800 | 676 |
| S26 | 19012690 | 41 | 18276868 | 41 | 735822 | 212971 | 166846 | 816 |
| S27 | 24748906 | 42 | 23771610 | 42 | 977296 | 359950 | 282750 | 27955 |
| S28 | 12802446 | 40 | 12511954 | 41 | 290492 | 122489 | 88974 | 3167 |
| S29 | 28504620 | 45 | 27635324 | 45 | 869296 | 484150 | 413354 | 1257 |
| S30 | 16010802 | 52 | 15377010 | 52 | 633792 | 439476 | 397370 | 4476 |
| S31 | 2830312 | 51 | 2615208 | 51 | 215104 | 58299 | 51161 | 1732 |
| S32 | 16181362 | 49 | 15867058 | 49 | 314304 | 563029 | 440953 | 106584 |
| S33 | 25791168 | 41 | 24088486 | 41 | 1702682 | 298290 | 229574 | 1092 |
| S34 | 24597504 | 40 | 23858136 | 40 | 739368 | 213471 | 161160 | 909 |
| S35 | 25772140 | 41 | 24698380 | 41 | 1073760 | 280428 | 212246 | 1422 |
| S36 | 24539818 | 40 | 24031958 | 40 | 507860 | 236172 | 174236 | 1214 |
| S37 | 37472534 | 41 | 35500286 | 41 | 1972248 | 488358 | 391914 | 4631 |
| S38 | 19983094 | 42 | 19023270 | 42 | 959824 | 256356 | 205391 | 5176 |
| S39 | 31091076 | 41 | 29898942 | 41 | 1192134 | 347081 | 270569 | 7394 |
| S40 | 17886242 | 43 | 17496682 | 43 | 389560 | 507385 | 460680 | 10331 |
| S41 | 16128590 | 44 | 15584176 | 44 | 544414 | 185184 | 150928 | 492 |
| S42 | 13308360 | 47 | 13000502 | 47 | 307858 | 1808465 | 1201966 | 632994 |
| S43 | 17158766 | 40 | 16813334 | 40 | 345432 | 183470 | 137056 | 744 |
| S44 | 36718566 | 39 | 36173204 | 39 | 545362 | 323779 | 241962 | 1384 |
| S45 | 38761104 | 44 | 36678314 | 44 | 2082790 | 988052 | 876523 | 13940 |
| S46 | 19889326 | 41 | 19468562 | 41 | 420764 | 210899 | 160243 | 1397 |
| S47 | 14860098 | 41 | 14366886 | 41 | 493212 | 187030 | 144521 | 8953 |
| S48 | 15767732 | 43 | 15255696 | 43 | 512036 | 191497 | 155094 | 567 |
| S49 | 13717748 | 44 | 13111944 | 44 | 605804 | 174458 | 137663 | 8622 |
| S50 | 16614480 | 41 | 15999454 | 41 | 615026 | 174597 | 126889 | 734 |
| NC1 | 31081106 | 39 | 30936344 | 39 | 144762 | 533474 | 216056 | 5421 |
| NC2 | 16572090 | 40 | 16526326 | 40 | 45764 | 336418 | 143650 | 6117 |
| NC3 | 53764818 | 38 | 53432216 | 38 | 332602 | 1152514 | 442565 | 13469 |
| NC4 | 55717858 | 40 | 55367686 | 40 | 350172 | 1056988 | 448162 | 12160 |
| NC5 | 20625390 | 39 | 20492572 | 39 | 132818 | 354243 | 145593 | 3728 |
| NC6 | 35663456 | 40 | 35417482 | 40 | 245974 | 681276 | 287498 | 7439 |
| NC7 | 18327126 | 45 | 18268248 | 45 | 58878 | 3859265 | 284427 | 1490220 |
| NC8 | 75306992 | 46 | 74920054 | 46 | 386938 | 3613504 | 2323483 | 891929 |
| **Total** | 1,353,368,514 |  | 1,315,842,908 |  | 37,625,606 | 32,674,936 | 20,156,856 | 4,718,443 |
| **Average** | 23,333,940 | 44 | 22,686,947 | 44 | 648,717 | 563,361 | 347,532 | 81,352 |
| **Median** | 20,701,371 | 42 | 20,282,963 | 42 | 528,225 | 324,707 | 235,211 | 2,838 |
| **Minimum** | 1,298,862 | 38 | 524,564 | 38 | 45,764 | 58,299 | 51,161 | 191 |
| **Maximum** | 55,717,858 | 94 | 55,367,686 | 94 | 2,082,790 | 3,947,451 | 2,258,473 | 1,490,220 |

**Supplementary Table 2.** Geographic location and year of collection data for each equine sample used in the study

| **Sample ID** | **Animal ID** | **State of origin of animal** | **Year collected** | **Prior diagnostic** |
| --- | --- | --- | --- | --- |
| S1 | 2940 | Victoria, Australia | 2016 | *Coxiella burnetii* |
| S2 | 2945 | Victoria, Australia | 2016 | *Coxiella burnetii* |
| S3 | 2951 | Victoria, Australia | 2016 | *Coxiella burnetii* |
| S4 | 2974 | Victoria, Australia | 2018 | *Coxiella burnetii* |
| S5 | 2197 | New South Wales, Australia | 2003 | *Coxiella burnetii* |
| S6 | 1397b | New South Wales, Australia | 1997 | *Coxiella burnetii* |
| S7 | 1355 | Victoria, Australia | 1997 | *Coxiella burnetii* |
| S8 | 2195 | Victoria, Australia | 2003 | *Coxiella burnetii* |
| S9 | 2220 | New South Wales, Australia | 2003 | *Coxiella burnetii* |
| S10 | 1886 | Victoria, Australia | 2002 | *Coxiella burnetii* |
| S11 | 1852 | Victoria, Australia | 2001 | *Chlamydia psittaci* |
| S12 | 2204 | New South Wales, Australia | 2003 | *Chlamydia psittaci* |
| S13 | 1995 | New South Wales, Australia | 2002 | *Chlamydia psittaci* |
| S14 | 1373 | Victoria, Australia | 1997 | *Chlamydia psittaci* |
| S15 | 1392 | Victoria, Australia | 1997 | *Chlamydia psittaci* |
| S16 | 2214 | New South Wales, Australia | 2003 | *Chlamydia psittaci* |
| S17 | 1606 | New South Wales, Australia | 2003 | *Chlamydia psittaci* |
| S18 | 2890 | Victoria, Australia | 2013 | *Chlamydia psittaci* |
| S19 | 2961 | Victoria, Australia | 2016 | *Chlamydia psittaci* |
| S20 | 2954 | Victoria, Australia | 2016 | *Chlamydia psittaci* |
| S21 | 1241 | New South Wales, Australia | 1996 | None |
| S22 | 2205 | New South Wales, Australia | 2003 | None |
| S23 | 2216 | New South Wales, Australia | 2003 | None |
| S24 | NEC | N/A (negative control) | - | Negative Control |
| S25 | 1429 | Victoria, Australia | 1998 | None |
| S26 | 1441 | Victoria, Australia | 1998 | None |
| S27 | 1445 | Victoria, Australia | 1998 | None |
| S28 | 1447 | Victoria, Australia | 1998 | None |
| S29 | 1360 | Victoria, Australia | 1997 | None |
| S30 | 1103 | Victoria, Australia | 1994 | None |
| S31 | 1164 | Victoria, Australia | 1994 | None |
| S32 | 1041b | Victoria, Australia | 1994 | None |
| S33 | 1550 | Victoria, Australia | 1999 | None |
| S34 | 1553 | Victoria, Australia | 1999 | None |
| S35 | 1564 | Victoria, Australia | 1999 | None |
| S36 | 1562 | Victoria, Australia | 1999 | None |
| S37 | 1569 | Victoria, Australia | 1999 | None |
| S38 | 1565 | Victoria, Australia | 1999 | None |
| S39 | 1571 | Victoria, Australia | 1999 | None |
| S40 | 1440 | New South Wales, Australia | 1998 | None |
| S41 | 1452 | New South Wales, Australia | 1998 | None |
| S42 | 1668 | Victoria, Australia | 2000 | None |
| S43 | 1664b | New South Wales, Australia | 2000 | None |
| S44 | 1558 | Victoria, Australia | 1999 | None |
| S45 | 2193 | Victoria, Australia | 2003 | None |
| S46 | 2196 | Victoria, Australia | 2003 | None |
| S47 | 2142 | Victoria, Australia | 2003 | None |
| S48 | 2190 | Victoria, Australia | 2003 | None |
| S49 | 2200 | New South Wales, Australia | 2003 | None |
| S50 | 2202 | New South Wales, Australia | 2003 | None |
| NC1 | NC1 | New South Wales, Australia | 2019 | Healthy birth |
| NC2 | NC2 | New South Wales, Australia | 2019 | Healthy birth |
| NC3 | NC3 | New South Wales, Australia | 2019 | Healthy birth |
| NC4 | NC4 | New South Wales, Australia | 2019 | Healthy birth |
| NC5 | NC5 | New South Wales, Australia | 2019 | Healthy birth |
| NC6 | NC6 | New South Wales, Australia | 2019 | Healthy birth |
| NC7 | NC7 | New South Wales, Australia | 2019 | Healthy birth |
| NC8 | NC8 | New South Wales, Australia | 2019 | Healthy birth |
